# Apparent Power Laws Can Occur without Criticality

**DOI:** 10.3390/e23111486

**Published:** 2021-11-10

**Authors:** Lawrence S. Schulman

**Affiliations:** Physics Department, Clarkson University, Potsdam, NY 13699-5820, USA; schulman137@gmail.com

**Keywords:** power laws, criticality, SOC

## Abstract

Power laws often lead to the conclusion that self-organized criticality is at work. This is not the case, and power laws can also occur away from criticality or can occur for other reasons.

Self-organized criticality (SOC) [1] answers many questions, in particular how a nongeneric condition appears to hold so often. This article, however, might be classed as contrarian, in that it points out that you do not have to be at criticality. To some extent, it is support for [2], who said that power laws (or distributions that resemble them) might not be power laws at all; in particular, the nonrarity of outliers (made more common in a power law) might not be accurate.

In this article, I will show that you can obtain data obeying power laws for several decades. One way is if you are near criticality; another has no relation to criticality (that I can discern). There are other ways as well.

First, we explore the concept of being near criticality.

I will start with the Ising model in two dimensions on a square lattice. At criticality ($k_{{}_{B}}T_{c}/J\approx 2.692$), it is known to have a power law in cluster size. See Figure 1a. However, far away, in the relative parameter space, at a temperature of three, you still obtain a power law. For the $600^{2}$ lattice, it does not encompass 4 or 4 1/2 decades, but 3 1/2, a match that is considered good enough to declare a power law.

Similarly, percolation is known to have a power law at criticality. However, for two-dimensional percolation, things are a bit tighter, but again, you do not need to be exactly at criticality. In Figure 2, I again show (approximate) criticality and a value close to, but not at, the value. Similar results hold for three-dimensional percolation—at least for forty-cubed arrays. See Figure 3. It should be noted the three-dimensional percolation is in a different universality class from the others. The three-dimensional Ising model (yet another universality class) has also been tested. See Figure 4. Here, only a temperature of 4.35 is shown. (The phase transition is at about a temperature of 4.545.) The reason is that at a high temperature, large clusters dominate: the three-dimensional percolation phase transition is at about 0.31, so that when the Ising temperature is large, the probability of 1/2 for spins up or down (which is the case at a high temperature) causes the approximately uncorrelated spins to form infinite clusters. Thus, we look *below* the transition temperature and look at the minority spins.

Other universality classes have not been checked. In particular, this applies to those phenomena related to purely quantum universality classes.

Data sets, which are often used to establish the existence of power laws, are necessarily limited. For example, the fractality of coastlines is generally limited to three or four decades of data. (They cannot do better, from the nature of the enterprise.) Moreover, sometimes, the first few entries cannot be trusted (as in the cities of New York and Los Angeles, whose populations do not follow the Zipf law): sometimes (for example, when there are more than three or four decades of data), the tail of the distribution needs a different slope, as found in word distributions [3]. There are a few really good straight lines, but they are limited to (at most) three or four decades (this comes up in neurology [4]). In practice, those figures—*not* at criticality—demonstrated are as good as the data sets that are presented in linguistics, cities, genes [5], and other areas.

Our thesis is thus not that we are witnessing SOC, but one of the mechanisms for obtaining power laws, a mechanism unrelated to SOC (see [6]) or (as will be explained below) no mechanism at all (or that I can think of). See also [7], where multiplicative noise is the origin, [8,9,10] where it is the sums of exponentials (see [11], Chap. 11 for an explicit example), as well as [12], where a particularly exotic power law appears. At best, one is *near* SOC, not *at* it, and clusters form naturally.

Then, there is the “other.” What I mean by this is the appearance of power laws for no apparent reason. I have in mind Huntley’s derivation of recombinant luminescence [13]. I will not review his work (it assumes reasonable properties, not always satisfied, but sufficiently often that one can obtain power laws), but provide a version of his graphs in Figure 5.

The *least* of the figures shows seven orders of magnitude (all logarithms are to base-ten). Is there a phase transition? Not that I can figure out. What Huntley has is a complicated integral, which gives approximately a power law.

There is phenomenon that brings you close to a critical point, but not all the way. I will illustrate it with a mean field example, but there is evidence that it is more general. This is feedback. If you look at [14], in engineering, feedback is mainly used to stabilize; a car going too slowly has cruise control, etc. However, in the physics literature [15], it has become natural to connect feedback with SOC (this is not to say that [14] did not deal with unstable systems, but that is not their emphasis).

Consider (N+1)-directed percolation. In the language of epidemiology (percolitis [16]), the directed percolation is a disease transmitted with probability r/N where *N* is the number of people and *r* a parameter. The mean field theory for the number of sick people is s(t+1)=1−exp(−s(t)r) (with *t*, time, measured in days). In equilibrium ($s_{0}\left(r\right)=s\left(\infty \right)$), this yields a critical value of r=1 and a behavior for r≥1 of $s_{0}\left(r\right)=2\left(r-1\right)$ (for *r* close to 1).

The feedback that is introduced here is immunity. Once a person has percolitis, that person is immune for *d* time units (days, in the example given). With *d* day immunity, the time-independent equation becomes 1−s=exp(−rs)+sd−sdexp(−rs). (This an application of Boolean variables, $A_{1}$ and $A_{2}$. In general $A_{1}|A_{2}=A_{1}+A_{2}-A_{1}A_{2}$. Here, $1-s=A_{1}|A_{2}$ is the probability of being healthy either because of failure to transmit or because of immunity.) The solution of the feedback equation, for *r* slightly above one, is:

(1) $$s=\frac{r-1}{d+1/2}\;.$$

This implies that *r* is effectively (for large *d*) divided by *d* in figuring the effect of this kind of feedback. The feedback thus brings one closer to the critical point, but still is not critical (this is seen explicitly in the galactic morphology application).

However, the role of feedback in becoming close to criticality is far more general. As examples, I cite [17,18,19,20,21]. In these articles, and others, there was an approach to criticality due to SOC, and as evidence, data were presented. However, I would say the data are at most three or four decades, and as to criticality, it is speculative to say that they will get there. Of course, there may be theoretical reasons to expect power laws (e.g., [22,23]), but as far as data are concerned, everything is finite.

As to whether or not the system is described by a power law, why not? This would be the ideal situation in which one is exactly at criticality, and since all data are approximate if the fit is good, you might as well make it a power law. However, along with that, I would want a bound on how far one is from criticality.

## Figures and Tables

**Figure 1:**
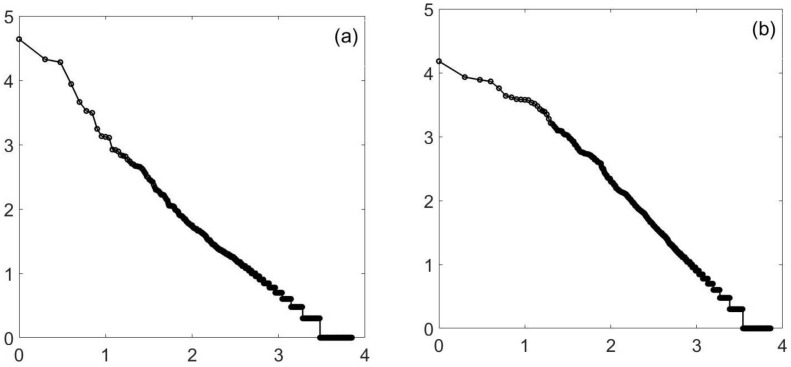
Ising, 2D. This is a log–log ($\log_{10}$) plot of the minority cluster distribution for the two-dimensional Ising model on a square lattice. It can easily pass for a straight line. A 600-by-600 lattice is used, and the slope is approximately −1. Plot (**a**) is at criticality, namely a temperature of 2.26919 with “*J*” =1. The second plot (**b**) is at a temperature of 3, more than 10% higher than the critical temperature. Again there, is a straight line, this time for (at least) 3 1/2 decades, enough for many authors to consider the power law proven. The slope is no longer −1, but is closer to −1.27.

**Figure 2:**
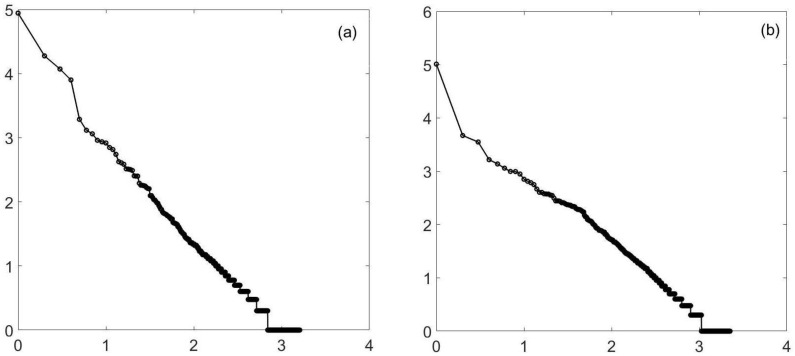
Percolation (simple), 2D. This is a log–log ($\log_{10}$) plot of the cluster distribution. It can easily pass for a straight line. A 500-by-500 2-dimensional square lattice percolation model is used, and the slope is approximately −1.2. Plot (**a**) is at criticality, a probability of 0.59. The second plot (**b**) uses a probability of 0.57. Again, there is a straight line, this time for (at least) 3 1/2 decades, enough for many authors to consider the power law proven. The slope is no longer −1.2, but is closer to −1.3.

**Figure 3:**
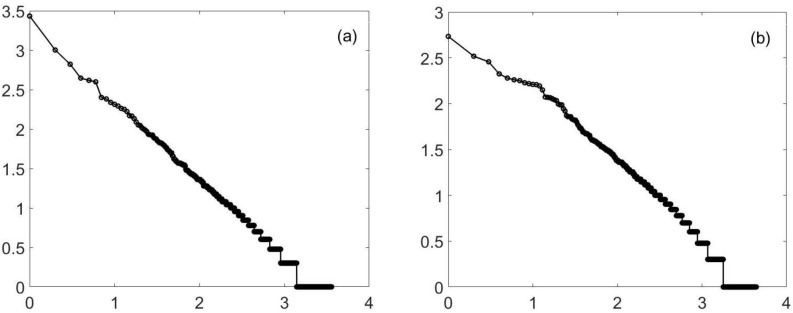
Percolation (simple), 3D. This is a log–log ($\log_{10}$) plot of the cluster distribution. It can easily pass for a straight line. A 40-by-40-by-40 3-dimensional hypercubic lattice percolation model is used, and the slope is approximately −0.93. Plot (**a**) is at criticality, a probability of 0.31. The second plot (**b**) uses a probability of 0.28 (a 10% difference). Again, there is a straight line, this time for (at least) 2 1/2 decades. The slope is no longer −0.93, but is closer to −0.92.

**Figure 4:**
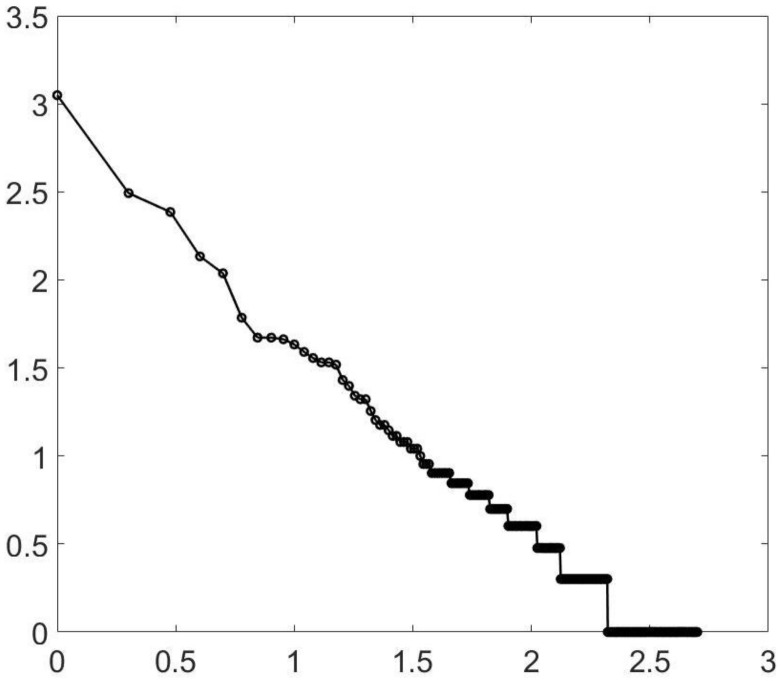
Ising, 3D. A log–log plot of the cluster distribution for a $25^{3}$ cubic lattice using Ising dynamics at a temperature of 4.35 (the criticality is about 4.55). This is below the transition and shows the minority spins. The line has a slope of approximately −0.9.

**Figure 5:**
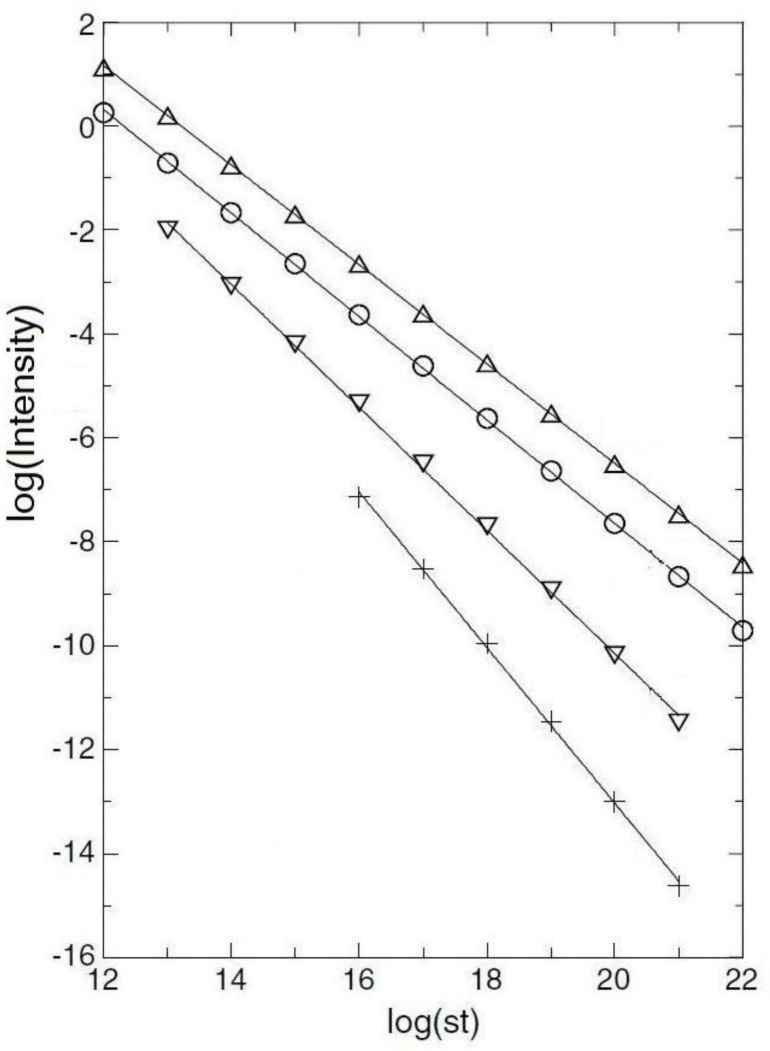
This shows in descending order various figures for reduced densities of traps. Reduced density $=\rho^{\prime }=\frac{4\pi }{3}\;\frac{\rho }{\alpha^{3}}$, with ρ the density of traps and α an effective tunneling rate. *s* is the “attempt frequency,” a quantity with dimensions of inverse time. *I* is the intensity of radiation. The reduced densities and slopes of the various curves are (symbol:) Δ: $\rho^{\prime }=2\times 10^{-6}$ and $I\propto t^{-0.958}$; ◯: $\rho^{\prime }=10^{-5}$ and $I\propto t^{-0.997}$; ▽: $\rho^{\prime }=5\times 10^{-5}$ and $I\propto t^{-1.185}$; +: $\rho^{\prime }=10^{-4}$ and $I\propto t^{-1.50}$.

## Data Availability

Not applicable.

## References

[1] Bak P., Tang C., Wiesenfeld K. (1987). Self-Organized Criticality: An Explanation of 1/*f* Noise. Phys. Rev. Lett..

[2] Stumpf M.P.H., Porter M.A. (2012). Critical Truth About Power Laws. Science.

[3] Montemurro M.A. (2001). Beyond the Zipf-Mandelbrot law in quantitative linguistics. Phys. A.

[4] Beggs J.M., Plenz D. (2003). Neuronal Avalanches in Neocortical Circuits. J. Neurosci..

[5] Harrison P.M., Gerstein M. (2001). Studying genomes through the aeons: Protein families, pseudogenes and proteome evolution. J. Mol. Biol..

[6] Newman M.E.J. (2005). Power laws, Pareto distributions and Zipf’s law. Contemp. Phys..

[7] Sornette D. (1998). Multiplicative processes and power laws. Phys. Rev. E.

[8] Bochud T., Challet D. (2007). Optimal approximations of power laws with exponentials: Application to volatility models with long memory. Quant. Financ..

[9] Goychuk I. (2009). Viscoelastic subdiffusion: From anomalous to normal. Phys. Rev. E.

[10] Palmer R.G., Stein D.L., Abrahams E., Anderson P.W. (1984). Models of Hierarchically Constrained Dynamics for Glassy Relaxation. Phys. Rev. Lett..

[11] Schulman L.S. (2021). When Things Grow Many: Complexity, Universality and Emergence in Nature.

[12] Pérez-Cárdenas F.C., Resca L., Pegg I.L. (2018). Coarse Graining, Nonmaximal Entropy, and Power Laws. Entropy.

[13] Huntley D.J. (2006). An explanation of the power-law decay of luminescence. J. Phys. Condens. Matter.

[14] Åström K.J., Murray R.M. (2008). Feedback Systems.

[15] Kadanoff L.P. (1991). Complex Structures from Simple Systems. Phys. Today.

[16] Schulman L.S., Seiden P.E. (1986). Percolation and Galaxies. Science.

[17] Buendía V., di Santo S., Bonachela J.A., Muñoz M.A. (2020). Feedback Mechanisms for Self-Organization to the Edge of a Phase Transition. Front. Phys..

[18] Byrd T.A., Erez A., Vogel R.M., Peterson C., Vennettilli M., Altan-Bonnet G., Mugler A. (2019). Critical slowing down in biochemical networks with feedback. Phys. Rev. E.

[19] Faggian M., Ginelli F., Marino F., Giacomelli G. (2018). Evidence of a Critical Phase Transition in Purely Temporal Dynamics with Long-Delayed Feedback. Phys. Rev. Lett..

[20] Liang J., Zhou T. (2018). Feedback-induced critical behavior in binary propagation on complex networks. Phys. Rev. E.

[21] Meisel C., Storch A., Hallmeyer-Elgner S., Bullmore E., Gross T. (2012). Failure of Adaptive Self-Organized Criticality during Epileptic Seizure Attacks. PLoS Comput. Biol..

[22] Helmrich S., Arias A., Lochead G., Wintermantel T.M., Buchhold M., Diehl S., Whitlock S. (2020). Signatures of self-organized criticality in an ultracold atomic gas. Nature.

[23] Urbach J.S., Madison R.C., Markert J.T. (1995). Interface Depinning, Self-Organized Criticality, and the Barkhausen Effect. Phys. Rev. Lett..

